# Pilot testing of an adaptive, individualized inhibitory control training for binge drinking: first evidence on feasibility, acceptance, and efficacy

**DOI:** 10.1007/s00426-022-01725-4

**Published:** 2022-08-22

**Authors:** Daniela Reichl, Niklas Enewoldsen, Astrid Müller, Sabine Steins-Loeber

**Affiliations:** 1 Department of Clinical Psychology and Psychotherapy, Otto-Friedrich University Bamberg, Markusplatz 3, 96047 Bamberg, Germany; 2 Department of Psychosomatic Medicine and Psychotherapy, Hannover Medical School, Hannover, Germany

## Abstract

**Background:**

Deficits in inhibitory control seem to promote habit behavior and therefore play an important role in the development and maintenance of addictive diseases. Although several training approaches have been suggested, there is a considerable lack of knowledge about the best way to improve inhibitory control. Based on a literature review regarding shortcomings of existing trainings, an individualized, adaptive inhibitory control training was developed. We aimed to assess feasibility and acceptance of this training and to provide preliminary results on its efficacy regarding inhibitory control and binge drinking.

**Methods:**

Sixty-one individuals (30 female) with binge drinking behavior were randomly allocated to either an experimental group receiving three sessions of the inhibitory control training or a waitlist control group receiving no training. Before and after the training, the participants performed a Go/NoGo task to assess inhibitory control (commission errors and false reaction time), completed a questionnaire on drinking-related self-control, and reported drinking behavior.

**Results:**

Although the training was feasible and accepted by participants, it did not affect self-control over drinking, inhibitory control or drinking behavior. The relationship between session number and false reaction time was linear for alcohol stimuli, but squared for neutral stimuli.

**Conclusion:**

Although our findings have to be interpreted in the light of some shortcomings, they demonstrate that further research is needed to enhance our understanding of how to improve inhibitory control and which factors might moderate this process.

**Supplementary Information:**

The online version contains supplementary material available at 10.1007/s00426-022-01725-4.

## Introduction

For the development of an addiction, the transition from goal-directed to habitual, automated behavior seems to be a relevant underlying mechanism (Everitt & Robbins, 2016; Lüscher et al., 2020). Individuals suffering from addiction show a hyperactivity of the bottom–up network, including, e.g., amygdala activity, while the antagonistic top–down network, including, e.g., prefrontal activity, is hypoactive. This makes it difficult for these individuals to inhibit reflexive, automated reactions, e.g., approach-behavior triggered by addiction-related cues (e.g., the respective substance; see Kozak et al., 2019). This deficit in the so-called inhibitory control (IC) seems to be an important predictor of relapse (Barreno et al., 2019; Czapla et al., 2016a). The I-PACE (Interaction of Person-Affect-Cognition-Execution) model (Brand et al., 2019), a model for the development of behavioral addictions, proposes that in early stages of addictive behavior, deficits in general IC, while in later stages, particularly problem-specific IC (i.e., in response to addiction-related cues) is associated with problem behavior. Deficits in IC can be shown in different experimental designs. For example, in respective computer tasks, a dominant motor response is created, e.g., by the instruction to press a key in response to certain visual stimuli, which has occasionally to be cancelled upon presentation of a signal (i.e. Stop-Signal paradigm) or withheld when stimuli of a different category are presented (i.e. Go/NoGo paradigm; see MacKillop et al., 2016). Failures to inhibit the response are interpreted as an indicator of deficits in IC, which have been demonstrated in several substance-related and behavioral addictions (Argyriou et al., 2017; Smith et al., 2014), as well as in individuals with risky drinking behavior (Carbia et al., 2018; Czapla et al., 2015; Henges & Marczinski, 2012). Strengthening top–down inhibitory control may lead to a better inhibition of the bottom–up network activity in favor of goal-directed behavior, and is therefore an important target of treatment interventions for addiction.

In general, computerized IC trainings are derived from the experimental assessment paradigms described above. In Table 1, we summarize the results and implications of previous studies.

**Table 1:** Results and implications of previous IC training studies

Study	Training condition (focused on problem behavior)	Control/comparison condition(s)	Session(s)	Problem behavior	Effect of training vs. comparison condition(s)		Suggestions for improvement of the training
					Proximal effects (on working mechanism)	Distal effects (on problem behavior)	
Di Lemma and Field (2017)	GNG (alcohol NoGo)	(1) Approach avoidance paradigm (2) GNG 50:50 ratio (Go:NoGo)	1	Heavy drinking		+ (Alcohol use in laboratory)	Test in a real-world setting, multiple sessions, combination with other interventions
Houben et al. (2011)	GNG (alcohol NoGo)	GNG alcohol Go	1	Heavy drinking	Devaluation of alcohol stimuli	+ (Self-reported alcohol use in daily life)	
Houben et al. (2012)	GNG (alcohol NoGo)	GNG alcohol Go	1	Heavy drinking	Devaluation of alcohol stimuli	+ (Alcohol use in laboratory & self- reported alcohol use in daily life)	Investigate more extensive paradigms
Kilwein et al. (2018)	GNG (alcohol NoGo)	GNG alcohol Go	1	Heavy drinking		+ (Alcohol use in laboratory and self- reported alcohol use in daily life)	More time to make decision, including hard liquor stimuli
Jones and Field (2013)	SST (alcohol Stop)	(1) SST neutral Stop (2) SST ignoring the Stop signal (3) SST only neutral cues	1	Heavy drinking	Improved inhibitory control (SST)	+ (Alcohol use in laboratory)	Modification of interventions to aim for long-term effects
Jones et al. (2018)	GNG (alcohol NoGo) and SST (alcohol Stop)	(1) SST only neutral cues (2) Picture categorization task	14	Heavy drinking			Develop improved treatment protocols, delivery on smartphones, individualization of stimuli, improving difficulty adaptation algorithms, enabling participants to form direct stimulus–reaction associations
Jones et al. (2020)	GNG (alcohol NoGo)	GNG 50:50 ratio (Go:NoGo)	2	Heavy drinking			Administration in a high-risk drinking environment, use of ecological momentary interventions
Smith et al. (2017)	1) GNG (alcohol NoGo) 2) SST (alcohol Stop)	(1) GNG only neutral cues (majority NoGo) (2) SST only neutral cues (3) Psycho-educative intervention	1	Drinking*			Individualization of stimuli, explicit instructions (respond to content of the pictures)
Strickland et al. (2019)	GNG (alcohol NoGo)	(1) Working memory task (2) Arithmetic problems	14	Alcohol Use Disorder	Improved inhibitory control (GNG)^a^	+ (Self-reported alcohol use in daily life)	Combination with working memory training, longer or more intensive interventions
Adams et al. (2017)	GNG (smoking NoGo)	GNG 50:50 ratio (Go:NoGo)	1	Smoking		+ (Inability to resist smoking)	Multiple sessions
Scholten et al. (2019)	SST (smoking Stop)	GNG smoking Go	1	Smoking	Devaluation of smoking stimuli		Multiple sessions, transformation into a video game
Scholten et al. (2021)	GNG (smoking NoGo)	Psycho-educative intervention	Minimum 5	Smoking	Devaluation of smoking stimuli		Combination with other interventions, use of game format
Bos et al. (2019)	GNG (smoking NoGo)	GNG smoking Go	14	Smoking			Combination with pharmacotherapy, Increasing the proportion of substance-related No-Go stimuli, individualization of stimuli, delivery on mobile devices
Hughes et al. (2021)	GNG (smoking NoGo)	GNG smoking Go	14	Smoking	Devaluation of smoking stimuli, reduced motivation to quit		Individualization of (control) stimuli, increasing the variability of the stimuli
Alcorn et al. (2017)	GNG (cocaine NoGo)	GNG only neutral cues	5	Cocaine Use Disorder			Multiple sessions
Rush et al. (2020)	GNG (cocaine NoGo)	GNG cocaine Go	18	Cocaine Use Disorder			Incorporation into other behavioral treatment approaches, more sessions, longer session duration, combination with other interventions (e.g., working memory training)
Verbruggen et al. (2012)	SST (during a gambling task)	Reaction task (press button when signal occurs during a gambling task)	1	Gambling*		+ (Gambling in laboratory after 2 h)	Use of problem-specific stimuli
Verbruggen et al. (2013)	SST (during a gambling task)	Reaction task (press button when signal occurs during a gambling task)	Experiment 1: 1 Experiment 2: 2	Gambling*		+ (Gambling in laboratory after 24 h)	Use of problem-specific stimuli
Stevens et al. (2015)	SST (during a gambling task)	Reaction task (press button when signal occurs during a gambling task)	1	Gambling*		+ (Gambling in laboratory)	Combination with other interventions

+ : positive effect (reduction of problem behavior) found

*IC*  inhibitory control, *GNG* Go/NoGo training, *SST* Stop-Signal training

^a^No control condition

*Participants were healthy volunteers

IC trainings in which participants practice to withhold reactions to substance-related stimuli (so-called Go/NoGo [GNG] trainings) seem to be more widespread and more effective (6 of 14 studies [42.9%] show positive effects on substance-related problem behavior; Table 1) than the Stop-Signal paradigm that trains the cancellation of motor responses (1 of 4 studies [25.0%] showed positive effects on substance-related problem behavior; Table 1). Likewise, several meta-analyses (Allom et al., 2016; Jones et al., 2016; Li et al., 2022) showed that the effect sizes of GNG trainings were larger than of Stop-Signal trainings (which were not significant) for cognitive functioning (e.g., inhibitory control, working memory) and different health behaviors (i.e., alcohol consumption and eating behavior). Furthermore, studies showed that the GNG paradigm may be more reliable than the Stop-Signal paradigm (Czapla et al., 2016b; Hedge et al., 2018). The better reliability and efficacy evidence is why the present study focused on GNG trainings.

Single-session GNG trainings have repeatedly been shown to reduce alcohol consumption in individuals with heavy drinking (Di Lemma & Field, 2017; Houben et al., 2011, 2012; Kilwein et al., 2018). These results are supported by a study investigating individuals with Alcohol Use Disorder (Strickland et al., 2019). Amongst smokers, IC trainings have led to an explicit devaluation of the trained pictures (Scholten et al., 2019), and showed positive effects on smoking behavior (Adams et al., 2017). However, there is also diverging evidence with regard to smoking behavior (Bos et al., 2019; Scholten et al., 2019). In other Substance Use Disorders (Alcorn et al., 2017; Rush et al., 2020), as well as in behavioral addictions, the evidence is very sparse (Luquiens et al., 2019). In different non-clinical samples, training IC led to reduced risk-taking in subsequent gambling tasks (Stevens et al., 2015; Verbruggen et al., 2012), even though the effects do not seem to be long-lasting (i.e., 24 h; see Verbruggen et al., 2013). Santiago et al. (2021) are currently conducting a study to evaluate a complex IC training program with different reaction tasks in problem gamblers.

From previous training studies, we also derived that an optimized training character may be required to produce stable and long-term effects (e.g., multiple sessions, individualization of the alcohol stimuli with regard to preferred drinks). From research on attentional bias tasks using substance stimuli, we know that individualized stimuli (e.g., preferred drink) can increase the reliability to a great extend (Christiansen et al., 2015). We are not aware of comparable research on IC tasks, but studies using individualized GNG tasks also reported good reliability findings (Czapla et al., 2016b). Additionally, research on attentional bias tasks showed that using individualized stimuli (with regard to one’s preferred alcoholic drink, e.g., wine, beer, vodka etc.), but not general alcohol stimuli (involving different drinks) were related to alcohol consumption in social drinkers (Christiansen & Bloor, 2014). Finally, adapting the task to the drinking behavior may increase the participants’ compliance. Previous studies using extended trainings are characterized by using multiple similar sessions (e.g., Jones et al., 2020; Scholten et al., 2021). However, individual adaptation of the difficulty may be useful to promote an overall training difficulty of moderate level (Benikos et al., 2013) and is therefore an important improvement for previous training paradigms (Peckham & Johnson, 2018). Furthermore, van Dessel et al. (2016) showed that, in a computer task that trains approach and avoidance reactions, awareness of the stimulus–reaction relation was a key factor for changes in stimulus evaluations. Against this background, we developed a novel training paradigm, meaning an individualized, adaptive explicit GNG paradigm training, in which participants are instructed to respond to a certain picture content (e.g., non-alcoholic drinks) while withholding their responses to a different content (e.g., alcoholic drinks), instead of reacting to a neutral cue like a colored picture frame. This may specifically address top–down IC in contrast to unconscious bottom–up processes. Being a more transparent task, this may also increase compliance. This is important, as Hughes et al. (2021) showed that a GNG training can also impede motivation to change. Even if Allom et al. (2016) showed no effect for training neutral stimuli, other research indicated that including neutral stimuli in the training protocol is reasonable (Smith et al., 2017), given that IC in reaction to both alcohol and neutral stimuli has shown to be predictive of a lower risk of relapse in alcohol addiction (Czapla et al., 2016a). Additionally, training IC regarding both neutral and substance-related stimuli seems to reduce craving and substance use problems (Hughes et al., 2021).

Interestingly, the working mechanism of GNG IC trainings is still unclear (Batschelet et al., 2020). There is neither enough evidence for the devaluation of substance-related stimuli, nor improved IC (Batschelet et al., 2020; Hughes et al., 2021). None of the previous studies investigating an alcohol-related GNG training paradigm (Di Lemma & Field, 2017; Houben et al., 2011, 2012; Kilwein et al., 2018) have systematically investigated the performance in withholding motor responses as outcome of interest. Even though Strickland et al. (2019) found an improved withholding performance after a 14-session training in individuals with Alcohol Use Disorder, they did not compare the withholding performance to their control groups (see also Jones et al., 2020). Only in smokers, Adams et al. (2017) investigated GNG performance, but found no effect. Overall, there is a considerable lack of studies investigating withholding performance as the mechanism of GNG IC trainings.

The present study sets out to assess feasibility and acceptance of this training. In addition, we provide preliminary results on its efficacy regarding the reduction of problematic drinking behavior as well as an increase in self-control, thereby examining the learning curve and the role of multiple sessions. We used a randomized controlled trial with two groups (training vs. no training). Given that the present study was designed as a controlled pilot study, we investigated a convenience non-clinical sample of adults reporting critical alcohol use as indicated by the Alcohol Use Disorder Identification Test (Babor et al., 2001) and binge drinking (BD), as a model of loss of inhibitory control.

## Methods

### Sample criteria

We recruited adults (≥ 18 years) screening positively on critical alcohol use during the last 6 months as indicated by the sum score (≥ 7 for women, ≥ 8 for men) in the Alcohol Use Disorder Identification Test (AUDIT; Babor et al., 2001). Furthermore, the participants had to fulfill the 4/5 criterion for BD during the last 6 months, namely four (women)/five (men) drinks per occasion (duration of 2 h) twice a month (Kilwein et al., 2018). Additionally, participants had to display behavioral BD characteristics during the last 6 months as indicated by a BD score ≥ 24 (Czapla et al., 2015) in the Alcohol Use Questionnaire (AUQ; Mehrabian & Russell, 1978). The questionnaires are further explained below (Assessment).

Exclusion criteria were a self-reported lifetime diagnosis of Substance Use Disorder, present diagnoses of psychiatric or neurological diseases, regular consumption of cannabis (at least once a month), occasional consumption of other drugs, or current intake of psychotropic medication. In the case of an AUDIT score ≥ 20, the participants received an information sheet on offers of help. Referring to previous training studies (e.g., Di Lemma & Field, 2017; Houben et al., 2012), we aimed at 30 participants for each study group.

### Procedure

Study advertisement took place via social media and flyers and a link to the online screening of the above-described inclusion and exclusion criteria was provided. To ensure the participants’ blindness to the study condition, both the training and assessment sessions were framed as “computer task” in the information sheets and instructions. Eligible participants were randomly assigned to an experimental group (EG), that completed three training sessions overall, or a passive control group (CG) without training. In the following pre-test, socio-demographic data, self-control, and IC were assessed. After a 10-min break, the first training session took place in the EG. In the following 10 days, two further training sessions were conducted in the EG summing up to three training sessions in total. Ten minutes following the last training session in the EG, or scheduled 10 days after the pre-test in the CG, a post-test to assess self-control and IC was administered. A follow-up was conducted 6–9 days after the post-test to assess the satisfaction with the training and drinking behavior. Assessments and training sessions were conducted online except for the first eight participants, who were included before the COVID-19 pandemic and tested in a university laboratory. A detailed guideline for the procedure was provided to the participants including reminders for every session. We collected time stamps to check if the participants correctly executed the procedure. The participants were instructed not to consume alcohol during the 10 h before the assessment and training sessions.

The study was approved by the Institutional Review Board. All participants provided written informed consent, and were optionally compensated with credit points or money.

### Training

The training was programmed with *python 3.8.3* (packages *psychopy 2020.1.3,* Peirce et al., 2019*; Scipy,* Virtanen et al., 2020; *Numpy,* Harris et al., 2020). The participants are instructed to react to Go stimuli (distractors) and inhibit their reaction to NoGo stimuli, depending on the content of the stimuli. Inhibition errors, namely responding to NoGo stimuli (so-called Commission Errors [CEs]), as well as a faster reaction time to NoGo stimuli (so-called false reaction time [FRT]) indicate deficits in response withholding.

Each of the three training sessions comprised two randomly presented categories: shapes (NoGo circles, Go rectangles) and pictures (NoGo alcoholic drinks, Go gardening tools). Each category comprised three blocks. In each block, 40 stimuli (majority were NoGo Stimuli with the exact contingency varying according to the adaptation algorithm, see below), placed on a 400*600 pixels white rectangle, were randomly presented on a black screen (inter-stimulus interval 1000 ms). After each block, a pause window occurred until key press. In sum, each training session consisted of six blocks (three shape blocks, three picture blocks) resulting in 240 presented stimuli. The difficulty of each session (Proportion of NoGo stimuli [NoGo rate] and presentation time) was variable (NoGo range: 5–35%, presentation time range: 350–1000 ms) depending on the performance in the previous session (algorithm based on Enge et al., 2014). The new presentation time was the mean reaction time in the previous session + 150 ms.

In case of good performance (low error rates in general), the difficulty increased: new NoGo rate = old NoGo rate – 0.025*(correct inhibitions in previous session/old NoGo rate).

In case of a bad performance (high error rates in general), the difficulty decreased: new NoGo rate = old NoGo rate + 0.025*(correct inhibitions in previous session/old NoGo rate).

For example, the NoGo rate of Person X was 30% (28 Go gardening stimuli, 12 NoGo alcohol stimuli) in training session 1. Person X made no commission errors (12 correct inhibitions), so the NoGo rate in training session 2 was 27.5% (29 Go gardening stimuli, 11 NoGo alcohol stimuli).

The difficulty of the first training session depended on the performance in the pre-test IC task (see Assessment IC computer task).

The stimuli were taken from four stimulus sets (alcoholic drinks, gardening tools, circles, and rectangles), each comprising 20 stimuli. For each training session and the IC assessment task (see Assessment IC computer task), 10 stimuli were chosen from each stimulus set, namely 10 alcoholic drinks, 10 gardening tools, 10 circles, and 10 rectangles. Thereby, we aimed for a balanced ratio. This means that all stimuli were presented with a similar frequency across blocks and training/task sessions. We also aimed for a comparable variation (i.e., in height, width, and color) of the shapes compared to alcoholic drinks and gardening tools.

The stimuli were individualized, such that the participants first selected their preferred sort of alcoholic drink from the choice of red wine, white wine, sparkling wine, dark beer, light beer, liquor, fruit-based spirits, cereal-based spirits, or other spirits. Pictures of this alcoholic drink were then presented throughout the training sessions. Furthermore, participants rated 30 gardening tools for their association with alcohol consumption. In the following, the 20 gardening tools with the lowest ratings were included in the gardening tools stimulus set.

After each block, a feedback screen with the percentage of correct responses (execution or withholding) occurred.

**Fig. 1:**
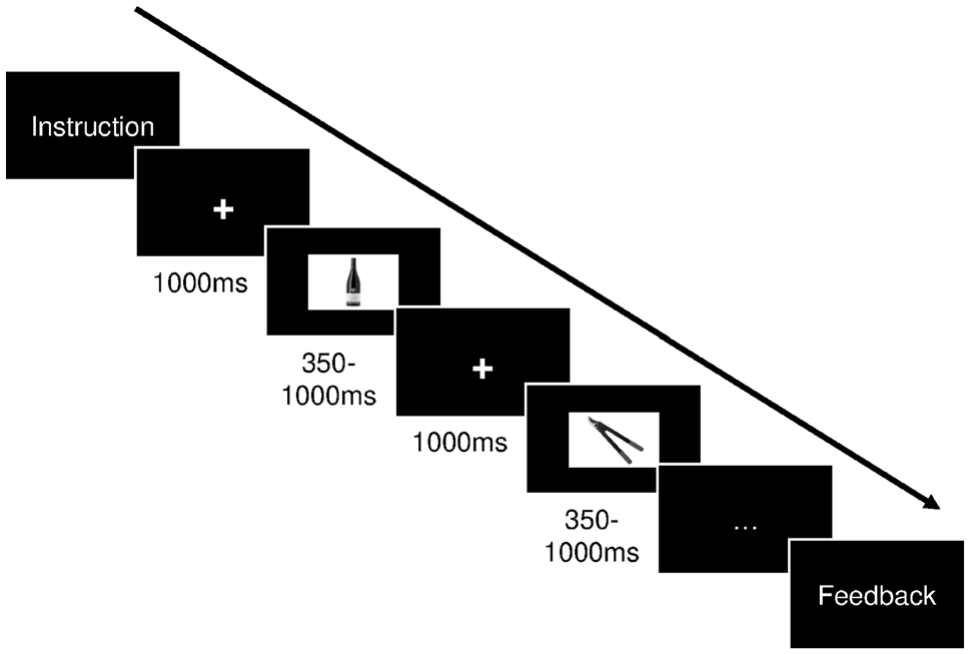
Example sequence of a training block

Figure 1 shows an example sequence of a training block.

### Assessment

#### Questionnaires

Socio-demographic data included self-reported age, sex (male vs. female), educational degree (none, lower secondary, secondary, higher, vocational training, university degree), and smoking (at least once a day; yes vs. no).

To screen for critical alcohol consumption, the AUDIT (Babor et al., 2001) was used. The sum score (range = 0–40, *α* = 0.66 in the present sample) of the ten items provides evidence for a risky drinking behavior (e.g., amount of alcohol, loss of control, and negative consequences). Scores of 7 (women)/8 (men) provide evidence for critical alcohol use. Scores of 16 and above indicate a high level of alcohol problems.

To screen for BD using the 4/5 criterion, we asked the participants how many times per month they have drunk at least four (women)/five (men) drinks on the same occasion over the course of the last 6 months. *At least two times per month* served as cut-off.

To screen for BD regarding behavioral characteristics, we calculated the AUQ (Mehrabian & Russell, 1978) BD score (Czapla et al., 2015). The last three items of the AUQ are combined as follows: 4* item 10 (number of drunk occasions) + item 11 (percentage of getting drunk when drinking) + 0.2* item 12 (average number of drinks per hour). The BD score, but regarding the past seven days, was also used as an outcome measure regarding the efficacy of the training.

Feasibility of the training was indicated by the login time stamps of the training and assessment sessions (e.g., to calculate the duration of each session and to examine, e.g., if the participants adhered to the 10-min break between assessment and first/last training session), as well as by the drop-out rates during the study.

Acceptance of the training was measured with an adapted version of the German Client Satisfaction Questionnaire (CSQ; Schmidt et al., 1989). The eight items (4-point Likert scale from 1–4) assess perceived quality, fulfillment of expectations, fulfillment of needs, recommendation to a friend, satisfaction with support, extend of support, overall satisfaction, and re-use of the training (range = 8–32; *α* = 0.91 in the present sample). Additionally, we provided an open question for unstructured feedback.

The Impaired Control Scale (ICS; Heather et al., 1993) provided information about deficits in self-control in the last week. Each item is rated on a 5-point Likert scale (strongly disagree [0]—strongly agree [4]). Three different subscales can be calculated. The first scale “Lack of intention to control drinking” (e.g., reversed “I have tried to limit the amount I drank”; *α* = 0.88 in the present sample; five items; range = 0–20), measures a different construct than the other two scales “Failures to control drinking” (e.g., “I have found it difficult to limit the amount I drank”; *α* = 0.70 in the present sample; ten items; range = 0–40) and “Perceived inability to control drinking” (e.g., “I would have difficulty limiting the amount I drank”; *α* = 0.81 in the present sample; ten items; range = 0–40).

Drinking behavior was assessed by the Timeline Followback method (TLFB, Sobell & Sobell, 1992) with regard to the past seven days. Participants were instructed to document their alcohol consumption (kind of alcohol and amount) on each day in a provided calendar. We then calculated the number of drinking days and the volume (grams of ethanol).

#### IC computer task

**Fig. 2:**
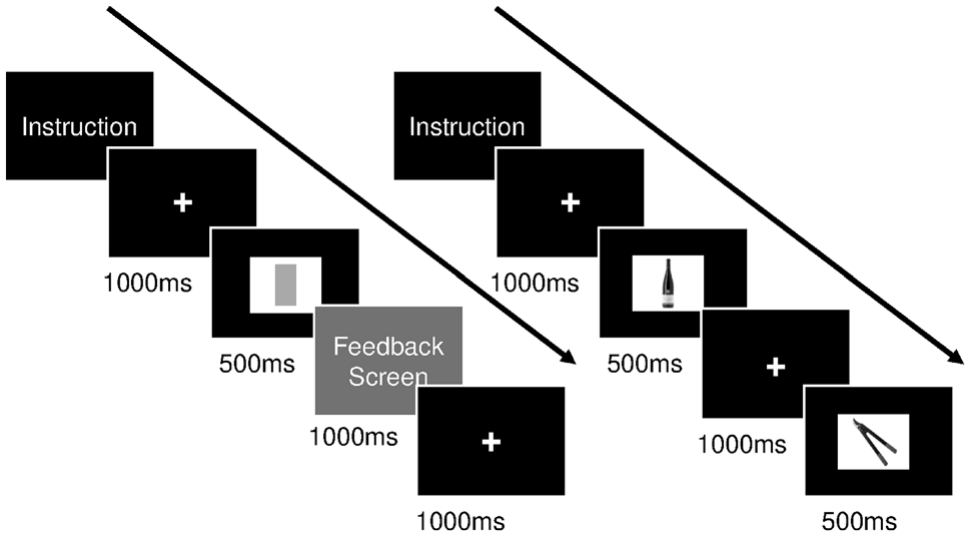
Example sequence of a practice (left) and task block (right)

The task to measure IC worked in the same way as the training, but with a practice block with visual feedback for every correct or wrong response and a steady NoGo rate (25%) and presentation time (500 ms). In each category, more CEs (range = 0–30; circles: *α* = 0.79; alcohol: *α* = 0.71) and a lower FRT (circles: *α* = 0.56; alcohol: *α* = 0.72) indicated deficits in response withholding. Figure 2 shows an example sequence of a practice block (left) and a task block (right).

### Data analysis

The data were edited and analyzed with *IBM SPSS Statistics 28* with a significance level of *α* = 5%. Given less than 5% missing values, only complete cases were analyzed.

With regard to feasibility, we report deviations from correct training execution, mean durations of the sessions, and drop-out rates.

With regard to acceptance, we describe the descriptive results of the CSQ (Schmidt et al., 1989), as well as the unstructured feedback.

With regard to the efficacy, we calculated mixed ANOVAs (lack of intention, CEs, FRT, BD score, drinking days, and volume) and a mixed MANOVA (failures and perceived inability; with follow-up mixed ANOVAs) to examine the effect of group (between-factor; EG vs. CG) and time (within-factor; pre vs. post/follow-up) with Bonferroni correction in post-hoc tests. If the scores’ distribution graphically deviated from a normal distribution (CEs, lack of intention, failures, BD behavior, drinking days, volume), we ran comparative analyses in *R*, i.e., a non-parametric model instead of ANOVA (library nparLD, Noguchi et al., 2012) and a Bayesian model for ordinal variables instead of MANOVA (ordered logistic, correlated population-level intercept and slope terms, multilevel random effect for participants and questions, custom implementation in Stan, Stan Development Team, 2019). We report deviations regarding the interpretation of the results. Additionally, repeated-measures ANOVAs/non-parametric Friedman tests were conducted to examine the learning curves, i.e., the effect of session number on relative CEs (= CEs/number of NoGo stimuli) and FRT. For all time*group interactions with at least small effects (*η*^2^ > 0.01), we conducted post-hoc power analyses.

In case of significant correlations of our outcomes with age (intention to control drinking) or sex (CEs, relative CEs), we conducted ANCOVAs controlling for age or sex, respectively. We will mention deviations with regard to the efficacy of the training and provide detailed results in Online Resource 1.

## Results

### Sample

**Fig. 3:**
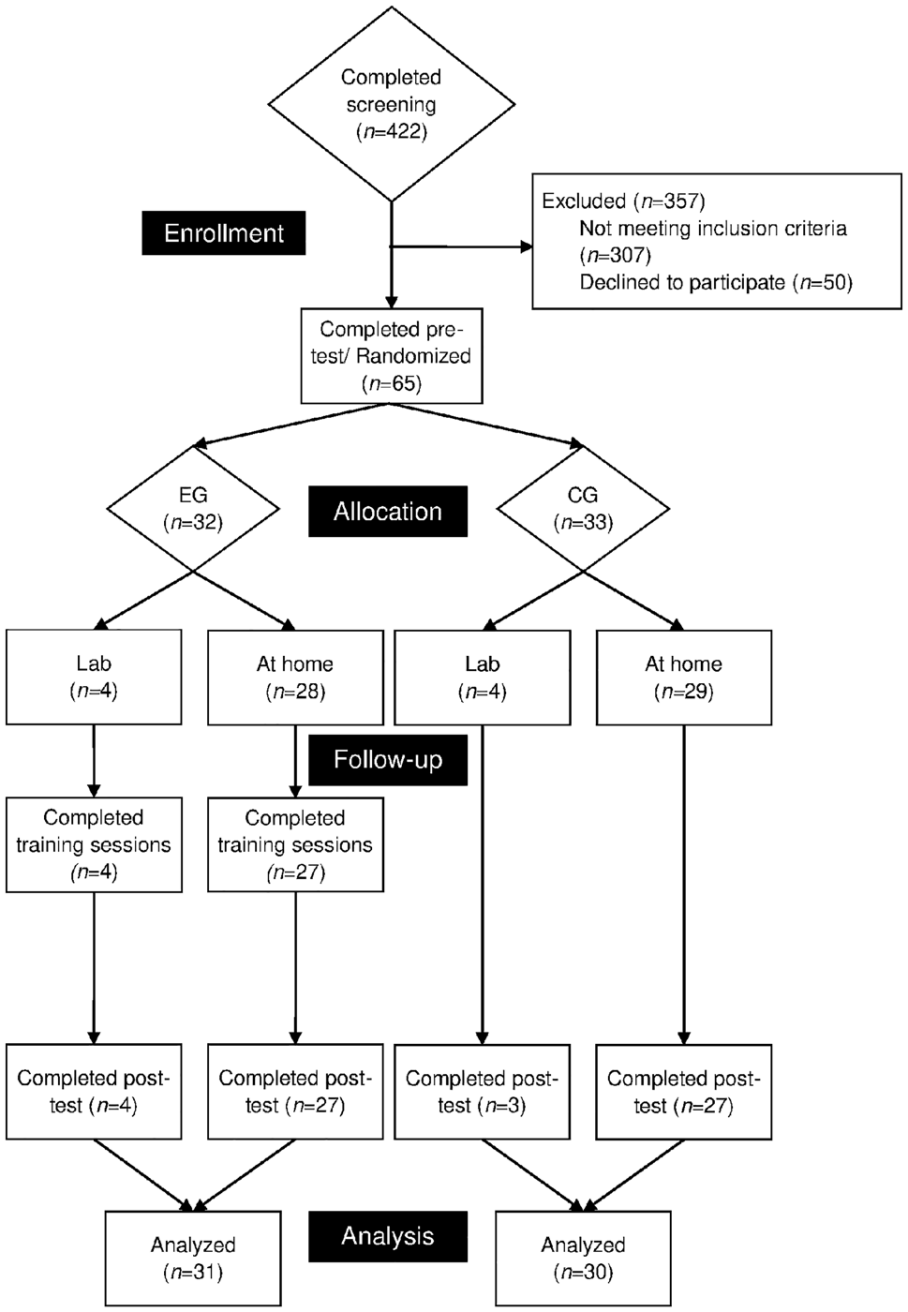
Study flow. *EG* experimental group, *CG* control group

The study was conducted from February to May 2020 (study flow in Fig. 3). Sixty-five participants were randomly allocated to the two group conditions. After randomization and pre-test, three participants dropped out of the study (CG: 2; EG: 1). For one participant in the EG, data of the IC task are missing due to technical problems. Thus, data from 61 participants could be analyzed.

The two study groups did not differ significantly with regard to demographic and drinking-related variables (Table 2). Both groups showed a high level of alcohol problems as indicated by the AUDIT score (Babor et al., 2001) and fulfilled the 4/5 criterion for BD on average more than four times a month.

**Table 2:** Socio-demographic and drinking-related variables

Variable	Training group	Control group	Statistics
Age [*M*(*SD*)]	24.71 (3.83)	26.03 (3.91)	*t*(59) = − 1.336, *p* = 0.187
Male sex [*n*(%)]	16 (51.61)	15 (50.00)	*χ*^2^(1) = 0.016, *p* = 1.000
Education [*n*(%)]			
Lower secondary	2 (6.45)	1 (3.33)	*χ*^2^(1) = 0.351, *p* = 1.000
Secondary	3 (9.68)	6 (20.00)	*χ*^2^(1) = 1.176, *p* = 0.472
Higher	22 (70.97)	24 (80.00)	*χ*^2^(1) = 0.373, *p* = 0.761
Vocational training	2 (6.45)	4 (13.33)	*χ*^2^(1) = 0.741, *p* = 0.671
University degree	16 (51.61)	16 (53.55)	*χ*^2^(1) = 0.000, *p* = 1.000
Smoking at least once a day [*n*(%)]	10 (32.26)	5 (16.67)	*χ*^2^(1) = 1.999, *p* = 0.235
Age of first alcohol consumption [*M*(*SD*)]	14.03 (1.11)	14.37 (1.13)	*t*(59) = − 1.166, *p* = 0.248
Binge score [*M*(*SD*)]	35.28 (10.22)	35.58 (9.67)	*t*(59) = − 0.119, *p* = 0.906
Frequency of 4/5 criterion [*M*(*SD*)]^a^	4.61 (0.84)	4.77 (0.94)	*t*(59) = − 0.675, *p* = 0.503
AUDIT total score [*M*(*SD*)]	16.32 (5.75)	15.90 (4.72)	*t*(59) = 0.313, *p* = 0.755

*AUDIT* Alcohol Use Disorder Identification Test

^a^Scale:1 = less than once a month, 2 = once a month, 3 = twice a month, 4 = three times a month, 5 = once a week, 6 = more than once a week

### Feasibility

Overall drop-out rate was 4.62% with no significant group differences (*χ*^2^(1) = 1.00, *p* = 1.00), indicating that the training condition was tolerated. In addition, the analysis of time stamps indicated that all participants correctly executed the training sessions, i.e., executing all sessions on the appointed date, adhering to the rest periods between sessions, no unusual rest periods during the sessions. Mean duration (only available for home participants) of a training session was 9.52 min (*SD* = 0.30).

### Acceptance

Acceptance ratings (Table 3) were of moderate height with regard to the possible score range.

**Table 3:** Descriptive acceptance of the training (CSQ subscales and total score)

Item	*M*	SD	Possible range
Perceived quality	2.90	0.70	1–4
Fulfillment of expectations	2.45	0.62	
Fulfillment of needs	2.23	0.62	
Recommendation to a friend	2.39	0.84	
Satisfaction with support	2.58	0.89	
Perceived extend of support	2.39	0.62	
Overall satisfaction	2.65	0.66	
Re-use of the training	2.26	0.97	
Total score	19.84	4.86	8–32

*CSQ* Client Satisfaction Questionnaire

Ten participants in the EG (32.26%) answered the open question on feedback. Positive feedback with regard to the training included statements on pleasure executing the training, the usefulness of the training, and the good technical execution (e.g., “The training was fun.”, “It was useful to train concentration and reaction time”, “It made my handling of alcohol more conscious”, “The technical execution and functioning of the training was described in detail; good technical execution.”, “It was easy to handle; the tasks were understandable and the structure of the training was simple.”).

Negative feedback focused on the questionable usefulness of the (computerized) training, boredom and loss of motivation to execute the task (“A computer training is unlikely to reduce alcohol abuse; it was not an alcohol therapy; I do not think that the training helped with regard to my drinking behavior.”, “The intended effect is not clear to me; I do not understand the mechanisms of the study/training.”, “The pictures were presented rather shortly so that one focuses more on the correct reaction rather than the alcohol cues; it was easier to react than not to react.”, “I did not like the task.”, “Sometimes it was a bit exhausting (to watch the shortly presented pictures and [not] to react); it was exhausting for my eyes and caused a headache.”, “In the first session, my motivation was very high, but very low in the other two sessions.”).

### Efficacy

The descriptive results of the variables of interest are displayed in Table 4. The main effects and interaction effects time*group are displayed in Table 5.

**Table 4:** Descriptive results of the variables of interest

Variable	Time point	Control group	Training group
Lack of intention	Pre	16.600 (4.272)	15.807 (5.269)
	Post	16.000 (4.363)	13.226 (5.789)
Inability	Pre	13.467 (6.388)	15.323 (5.724)
	Post	10.833 (5.657)	11.936 (5.079)
Failures	Pre	11.967 (5.945)	12.226 (6.607)
	Post	10.567 (6.328)	9.000 (6.522)
False reaction time (ms) shapes	Pre	0.396 (0.025)	0.384 (0.029)
	Post	0.398 (0.032)	0.367 (0.031)
False reaction time (ms) alcohol	Pre	0.370 (0.042)	0.374 (0.036)
	Post	0.383 (0.053)	0.390 (0.036)
Commission errors shapes	Pre	9.970 (4.951)	10.060 (4.442)
	Post	9.570 (5.049)	10.030 (5.834)
Commission errors alcohol	Pre	5.030 (2.895)	5.940 (4.494)
	Post	5.200 (4.097)	6.610 (5.766)
Drinking days	Pre	2.067 (1.760)	2.323 (1.887)
	Follow-up	2.367 (1.586)	1.839 (1.695)
Alcohol volume (gr)	Pre	135.230 (154.066)	125.987 (135.031)
	Follow-up	151.207 (170.261)	127.565 (147.896)
Binge drinking score	Pre	17.513 (31.662)	15.632 (26.175)
	Follow-up	19.963 (31.453)	12.697 (28.219)

**Table 5:** Main effects and interaction effects time*group

Variable	Effect	*df*	Statistic	*p*	*η*^2^
Lack of intention	Time	1.59	9.317	0.003	0.136
	Group	1.59	2.355	0.130	0.038
	Time*Group	1.59	3.613	0.062	0.058^a^
Inability	Time	1.59	22.636	< 0.001	0.277
	Group	1.59	1.250	0.268	0.021
	Time*Group	1.59	0.355	0.554	0.006
Failures	Time	1.59	5.110	0.027	0.080
	Group	1.59	0.266	0.608	0.004
	Time*Group	1.59	0.796	0.376	0.013^b^
Commission errors	Part	1.59	88.784	< 0.001	0.601
	Time	1.59	0.048	0.828	0.001
	Group	1.59	0.588	0.446	0.010
	Time*Group	1.59	0.217	0.643	0.004
	Time*Group*Part	1.59	0.006	0.936	< 0.001
False reaction time (ms)	Part	1.57	2.278	0.137	0.038
	Time	1.57	0.376	0.542	0.007
	Group	1.57	1.367	0.247	0.023
	Time*Group	1.57	0.596	0.443	0.010
	Time*Group*Part	1.57	3.124	0.083	0.052^c^
Drinking days	Time	1.59	0.160	0.691	0.003
	Group	1.59	0.128	0.722	0.002
	Time*Group	1.59	2.908	0.093	0.047^d^
Volume (gr)	Time	1.59	0.172	0.680	0.003
	Group	1.59	0.253	0.617	0.004
	Time*Group	1.59	0.116	0.735	0.002
Binge drinking behavior	Time	1.59	0.117	0.733	0.002
	Group	1.59	0.306	0.582	0.005
	Time*Group	1.59	0.055	0.816	0.001

^a^Post hoc power: 96.9

^b^Post hoc power: 29.3

^c^Post hoc power: 97.4

^d^Post hoc power: 83.7

#### Self-reported self-control over drinking

For the subscales failures and inability, the MANOVA revealed no interaction time*group, *F*(2,58) = 0.396, *p* = 0.675, *η*^2^ = 0.013, but a significant main effect of time, *F*(2,58) = 11.312, *p* < 0.001, *η*^2^ = 0.281, indicating that all participants reported less impairment of self-control from the pre-test to the second follow-up. Results of the follow-up ANOVAs for these two scales are displayed in Table 5. The ANOVA’s main effects of time indicated that the lack of intention, failures, and perceived inability to control drinking decreased from pre- to post-test, but independently of the study group. Using non-parametric tests, the interaction time*group for lack of intention was close to significant (*p* = 0.051), with continuity-corrected Wilcoxon signed rank tests indicating that the lack of intention decreased in the EG, *V* = 249, *p* = 0.005, but not in the CG, *V* = 134.5, *p* = 0.517.

#### Deficits of inhibitory control

A main effect of category in the ANOVA indicated that the participants made more CEs in reaction to circles (vs rectangles), *M* = 9.910, *SD* = 4.112, than in reaction to alcohol (vs gardening tools), *M* = 5.705, *SD* = 3.978, *p* < 0.001. Other than that, no main or interaction effects including time*group were significant, indicating no significant changes in IC.

#### Learning curve of inhibitory control

With regard to shape stimuli, we found no effect of time on relative CEs, *F*(4,120) = 1.610 *p* = 0.176, *η*^2^_partial_ = 0.051, but an effect of time on FRT, *F*(4,116) = 8.180, *p* < 0.001, *η*^2^_partial_ = 0.220. The squared contrast was significant, *F*(1,29) = 18.787, *p* < 0.001, *η*^2^_partial_ = 0.393. Pairwise comparisons indicated that FRT to circles tended to increase, *p* = 0.056, from pre-test to training session 2, and then decreased to post-test, *p* = 0.006.

**Fig. 4:**
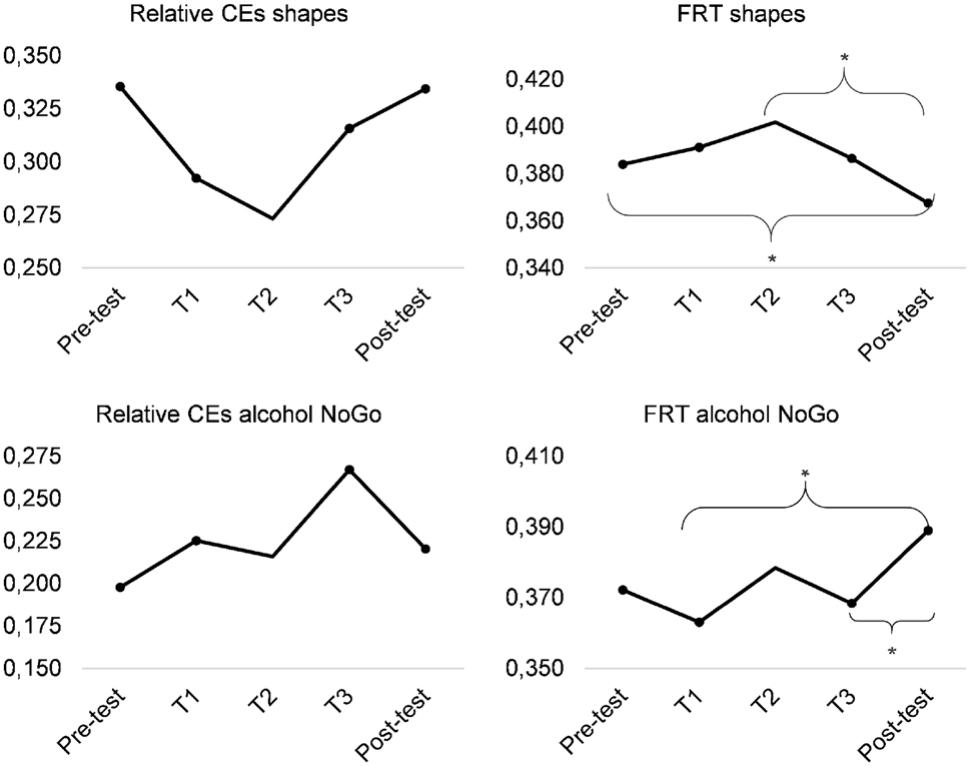
Changes of inhibitory control over the course of the training sessions. Relative *CEs* relative commission errors, *FRT* false reaction time, *T* training session

With regard to alcohol stimuli, we found no effect of time on relative CEs, *F*(4,120) = 1.593, *p* = 0.180, *η*^2^_partial_ = 0.050, but an effect of time on FRT, *F*(4,112) = 2.750, *p* = 0.032, *η*^2^_partial_ = 0.089. Pairwise comparisons indicated that FRT to alcohol stimuli increased from training session 1 and 3 to the post-test, respectively, *p* = 0.009 and 0.028. The linear contrast was significant for FRT, *F*(1,28) = 4.384, *p* = 0.045, *η*^2^_partial_ = 0.135. Figure 4 shows the changes of relative CEs and FRT over the course of the training sessions (T1-3).

#### Drinking behavior

No main and interaction effects were observed in the ANOVAs for BD score, drinking days, and volume, indicating no changes in drinking behavior.

## Discussion

The present pilot study assessed feasibility and acceptance of an optimized version of a computerized GNG training according to implications of previous research and provides pilot data on its efficacy regarding inhibitory control and BD. To provide a deeper understanding of the impact of every training session, we also analyzed the learning curve regarding IC.

The results support the feasibility of the training, even if conducted as an online intervention. The participants correctly executed the procedure. The drop-out after study inclusion was low.

Although the participants in the present study showed a sufficient degree of acceptance, they mentioned a lack of transparency, which may impede compliance. Thus, the explicit training format may have not fulfilled its purpose.

Our pilot investigation showed that, independently of the study group (CG vs. EG), all participants reported a decrease in the perceived inability and failures to control drinking. The time *group interaction effects did not achieve significance, although descriptive data indicated larger improvements for the EG. Thus, it can be hypothesized that the training may have improved self-reported self-control, but this was not demonstrated in the present study due to a lack of power. While non-parametric analysis suggested that the training might also lead to increased attention of the participants to their drinking behavior, this again has to be interpreted with caution given that the interaction just missed significance. Given that only small beneficial effects were observed, if at all, it can thus be assumed that the clinical relevance of these effects is not given, especially as no effects on drinking were observed. The lacking effects on drinking behavior contradict a few other studies showing an effect of GNG trainings on self-reported drinking behavior outside the laboratory. However, these studies used either an antagonistic control group (i.e., training to react to alcohol stimuli, but not neutral stimuli; Houben et al., 2011, 2012; Kilwein et al., 2018), which may artificially increase the effect of the training, or investigated individuals with an Alcohol Use Disorder (Strickland et al., 2019), who may have a higher motivation to change than individuals with non-pathological BD. Additionally, Strickland et al. (2019) provided more sessions than the present study. Studies with heavy drinkers that investigated more conservative control groups found only an effect on immediate drinking behavior in the laboratory (Di Lemma & Field, 2017; Jones & Field, 2013), or no effect (Jones et al., 2018, 2020).

Interesting conclusions can be derived from the analysis of the learning curves regarding IC. While there was no overall effect of the training with regard to IC (CEs and FRT), the learning curves revealed a non-linear relationship between session number and IC (in terms of higher FRT). The performance peak for shape stimuli in training session 2 leads to the conclusion that only the first training session had a positive effect on subsequent performance, while the other training sessions worsened IC. Regarding alcohol stimuli, we found IC (FRT) to increase over the sessions. Here, both the first and third training session had a positive effect on IC, and it remains an open question if more than three adaptive training sessions could exceed the effect of the first session. In addition, it may be that the learning curve is characterized by ups and downs due to factors moderating or mediating the efficacy of the training. For example, participants’ mood during the training may affect inhibitory control as previously demonstrated for food-related inhibition (Loeber et al., 2018). Furthermore, the exposure to the alcohol-related stimuli during the training may induce craving which in turn might affect inhibition. Only recently, a study demonstrated an association between impulsive behavior and craving in Alcohol Use Disorder which is mediated by emotion regulation competencies (Reichl et al., 2022). Thus, there may be a complex interaction of these processes during IC trainings, and future studies are warranted to further investigate the working mechanism of such trainings. For behavioral addictions, similar interacting effects of craving, cue-reactivity, and inhibitory control are proposed in the I-PACE model (Brand et al., 2019).

The finding that individuals with BD behavior showed more deficits in withholding reactions to circles stimuli than to alcohol stimuli is somewhat surprising and contradicts previous alcohol-related IC studies (Czapla et al., 2015, 2016a). This may be due to lower alcohol-related IC deficits in our study.^1^ Importantly, we used gardening tools as Go stimuli/distractors in the alcohol No-Go category, while previous studies (Czapla et al., 2015, 2016a) used non-alcoholic drinks as distractors. This may also have altered the likelihood of commission errors, which can be derived from food-specific IC studies (Meule, 2017). We decided against non-alcoholic drinks, given that they may be associated with alcohol consumption (e.g., regarding long drinks) and, therefore, are not a suitable control category. Additionally, the probability of NoGo stimuli was higher in the present study (25% instead of 20%), which makes successful inhibition more likely (Wessel, 2018). However, this difference seems negligible given that IC performance regarding shapes was comparable to other studies.

## Practical conclusions for training inhibitory control

Given that the participants of our study completed the training sessions as scheduled, and drop-out in the training group was low and not significantly different from the control group, we conclude that a computerized online training to improve self-control is in general a viable treatment approach. However, the training suggested here needs to be adapted to improve its efficacy and to achieve clinical significant changes. The results of the learning curve analysis, namely that three sessions lead to unstable training effects, suggest to investigate the efficacy of a larger number of adaptive sessions, e.g., six sessions as suggested for other computer training paradigms (Eberl et al., 2014). However, one participant reported that the motivation dropped after the first training session. Another participant’s feedback suggests that this could be explained by the sessions being rather exhaustive. Thus, including more breaks could be reasonable. Additionally, the clarification of the purpose and the mechanisms of the training may increase transparency and, thus, motivation for the training.

## Implications for future research

As outlined in the introduction, the development of addiction can be described as a process in which drug seeking, or other behaviors like gaming or shopping, become more and more habitual and less goal-directed. Cues that have often been associated with the behavior (e.g., the sight and smell of alcohol, or a certain shopping website; Trotzke et al., 2020; Vogel et al., 2019), can induce changes of attention allocation, impairment of inhibitory control, and finally conditioned habitual responding (e.g., alcohol consumption or buying of unnecessary items). Similar processes are assumed to play a central role in a number of other disorders and response inhibition trainings have been evaluated for example for Binge Eating Disorder and obesity. Regarding food-related deficits of response inhibition, several studies demonstrated positive effects on food-intake and weight loss (e.g., Houben & Jansen, 2011; Lawrence et al., 2015; for a review, see Jones et al., 2016). In contrast, as outlined in the introduction, regarding heavy drinking and Alcohol Use Disorder, findings are more inconsistent, while no effects could be demonstrated for Nicotine Use Disorder or Cocaine Use Disorder. These findings suggest that improving response inhibition may be more challenging for drugs of abuse compared to natural rewards and may be related to dopamine signaling in brain reward circuitry (Bamford et al., 2018). For future research aiming to improve the treatment of disorders related to appetitive habitual behavior, it seems important to enhance our understanding of how basic mechanisms of reward processing and response inhibition are altered in different mental disorders and to derive more tailored interventions. For example, a recent study by Dormal and colleagues (Dormal et al., 2020) suggested that combining the GNG paradigm with transcranial direct-current stimulation may be a promising way to improve IC in BD as it was shown to promote attention-related brain activity. In addition, Bouton (2021) recently reviewed a number of animal studies which demonstrate that a habit can be returned to a goal-directed action, for example by a context switch or pharmacological interventions. However, there is at present a scarcity of experimental studies in humans to enhance our understanding how habit behavior in addiction can be addressed apart from increasing response inhibition or extinguishing cue-conditioned responses, both of which are of limited effectiveness (Jones et al., 2016; Mellentin et al., 2017).

## Limitations

The results have to be interpreted in the light of some limitations. Although comparable to previous IC studies (e.g., Di Lemma & Field, 2017; Houben et al., 2012), the sample size of our study was rather small. Notably, subjective compared to objective measurements, as well as follow-up rather than immediate assessments produce smaller effect sizes and, thus, require larger samples (Allom et al., 2016). For instance, we may have not had enough power to detect the small interaction time*group regarding failures to control drinking. However, a small effect size may not be clinically relevant.

We also did not include an active control group. Previous studies compared for example the training condition of interest to a training in the opposite direction (e.g., alcohol Go) which might artificially increase the effect of the intervention. In contrast, we wanted to examine if there is any effect of our novel (explicit, adaptive, individualized, including neutral and problem-related stimuli) training paradigm in individuals with BD, before comparing the training to other approaches. However, we, thus, cannot rule out that simply spending more time in the study influenced self-reports in the EG. Future studies could, e.g., include a categorization task as active control group.

Additionally, the study was conducted at the beginning of the Covid-19 pandemic, which could have distorted the results. The majority of the participants was included after the beginning of the lockdown. The restrictions have shown to increase heavy drinking (Irizar et al., 2021; Kilian et al., 2022). On the other hand, studies provide evidence for a reduction in BD (Manthey et al., 2020). Overall, the pandemic may have influenced the drinking behavior in the German population (increase or reduction, depending on the drinking pattern), which may have changed the likelihood to observe an effect in the present study. However, we do not expect a distorting effect on the behavioral IC measure.

At last, while habit was assumed to be the mechanism that the present IC training was directed towards, this assumption was not tested directly, for example by administering a Pavlovian to instrumental Transfer task which aims to assess the effect of conditioned stimuli on instrumental responding (e.g., Steins-Loeber et al., 2020).

## Conclusions

Overall, we were the first to investigate both objective changes in response withholding and subjective self-control, which previous studies on GNG IC training did not take into account (Batschelet et al., 2020). Explicitly training to withhold motor responses in a computer task with three individualized adaptive sessions may not improve top–down IC. Thus, future studies should address different routes to address BD.

## Supplementary Information

Below is the link to the electronic supplementary material.

Supplementary file1 Results of the ANCOVAs regarding the effect of time and/or group after controlling for sex or age (PDF 201 KB)

## Data Availability

The dataset is available in the OSF Registries repository under https://osf.io/fcgmr/.

## Footnotes

1 The CE proportion regarding shapes in the present study (9.91/30 = 0.33) was comparable to Czapla et al., (2016a; alcohol dependence: 0.36, healthy controls: 0.28) and Czapla et al., (2015; individuals with BD: 0.41), while the CE proportion regarding alcohol (5.71/30 = 0.19) was lower than in these studies (alcohol dependence: 0.43, healthy controls: 0.33, individuals with BD: 0.59).
