# Supplementary material for: Pilot testing of an adaptive, individualized inhibitory control training for binge drinking: first evidence on feasibility, acceptance, and efficacy
Source: Psychol Res. 2022 Aug 22;87(4):1267–83. doi: 10.1007/s00426-022-01725-4 (PMC9395788; doi:10.1007/s00426-022-01725-4)
Supplement: Supplementary file 1 — Supplementary file1 Results of the ANCOVAs regarding the effect of time and/or group after controlling for sex or age (PDF 201 KB) [file 426_2022_1725_MOESM1_ESM.pdf]

Pilot testing of an adaptive, individualized inhibitory control training for binge drinking: first evidence on feasibility, acceptance, and efficacy

Daniela Reichl[1], Niklas Enewoldsen[1], Astrid Müller[2], Sabine Steins-Loeber[1]

[1] Department of Clinical Psychology and Psychotherapy, Otto-Friedrich University Bamberg, Bamberg, Germany

[2] Department of Psychosomatic Medicine and Psychotherapy, Hannover Medical School, Hannover, Germany

Journal: Psychological Research

Address of correspondence:

Daniela Reichl

Otto-Friedrich University Bamberg

Markusplatz 3

96047 Bamberg

GERMANY

Email: daniela.reichl@uni-bamberg.de.

ESM1. Results of the ANCOVAs regarding the effect of time and/or group after controlling for sex or age

For commission errors, the ANCOVA revealed no interaction time*sex, $F(1,57)=0.780$, $p=.381$, $\eta^2=.013$, of group*sex, $F(1,57)=0.072$, $p=.789$, $\eta^2=.001$, time*group*sex, $F(1,57)=0.247$, $p=.621$, $\eta^2=.004$, or time*group*sex*part, $F(1,57)=0.904$, $p=.346$, $\eta^2=.016$. The effects of group, $F(1,57)=0.660$, $p=.420$, $\eta^2=.011$, time, $F(1,57)=0.057$, $p=.812$, $\eta^2=.001$, and time*group, $F(1,57)=0.234$, $p=.630$, $\eta^2=.004$, and time*group*part, $F(1,57)=0.003$, $p=.954$, $\eta^2=.000$, did not change.

For relative commission errors regarding shapes, the ANCOVA revealed no interaction time*sex, $F(4,116)=0.554$, $p=.696$, $\eta^2=.019$. The effect of time, $F(4,116)=1.581$, $p=.184$, $\eta^2=.052$, did not change.

For relative commission errors regarding alcohol, the ANCOVA revealed no interaction time*sex, $F(4,116)=0.746$, $p=.563$, $\eta^2=.025$. The effect of time, $F(4,116)=1.626$, $p=.172$, $\eta^2=.053$, did not change.

For the intention to control drinking, the ANCOVA revealed no interaction time*age, $F(1,58)=0.530$, $p=.470$, $\eta^2=.009$. The effects of group, $F(1,58)=3.750$, $p=.058$, $\eta^2=.061$, and time*group, $F(1,58)=3.029$, $p=.087$, $\eta^2=.050$, did not change. Only, the effect of time was no longer significant, $F(1,58)=1.371$, $p=.246$, $\eta^2=.023$.
